# Assessing Measurement Repeatability of a Novel Anisotropic Phantom for Advanced Diffusion MRI Models

**DOI:** 10.1002/mrm.70330

**Published:** 2026-03-06

**Authors:** Lauren Stephens, Sofia Chavez, Fergal Kerins, Michael D. Noseworthy

**Affiliations:** ^1^ McMaster School of Biomedical Engineering McMaster University Hamilton Ontario Canada; ^2^ Imaging Research Centre, St. Joseph's Healthcare Hamilton Ontario Canada; ^3^ Department of Medical Imaging McMaster University Hamilton Ontario Canada; ^4^ PreOperative Performance Toronto Ontario Canada; ^5^ Department of Electrical and Computer Engineering McMaster University Hamilton Ontario Canada

**Keywords:** anisotropic phantom, constrained spherical deconvolution, diffusion kurtosis imaging, diffusion MRI, diffusion tensor imaging, quality assurance

## Abstract

**Purpose:**

Diffusion MRI is widely used to characterize tissue microstructure, but standardization remains challenging, particularly for advanced models or regions with crossing fibers. Phantoms provide controlled environments to assess measurement repeatability independent of biological variability. This study evaluated the repeatability of higher‐order diffusion tensor metrics using a novel anisotropic diffusion phantom designed to mimic white matter tract geometry.

**Methods:**

The phantom, containing linear, crossing (30°, 45°, 90°), and bifurcating synthetic fiber bundles, was scanned seven times using a GE Healthcare 3.0 T MRI system. Four acquisition protocols were evaluated: 30‐direction DTI (*b* = 1000s/mm^2^), 60 and 90‐direction High Angular Resolution Diffusion Imaging (HARDI; *b* = 1300s/mm^2^), and 30‐direction Diffusion Kurtosis Imaging (DKI; *b* = 250, 500, 750, 1000, 1500, 2000, 2500, 3000 s/mm^2^). Repeatability was quantified using coefficient of variation (CoV) and intraclass correlation coefficient (ICC) for scalar diffusion metrics across six regions of interest. Fiber orientation distribution functions (fODFs) were analyzed to assess crossing fiber resolution accuracy.

**Results:**

DTI‐derived metrics demonstrated excellent repeatability, with fractional anisotropy (FA) CoV < 10% and mean, axial, and radial diffusivities < 3%. DKI‐derived metrics exhibited greater variability, though kurtosis FA remained stable (CoV ∼7%). Generalized FA showed improved reliability with increased angular resolution (ICC = 0.8445 for 90‐direction HARDI). fODFs accurately resolved crossing fibers at 90° (RMSE = 3.49°) and 45° (RMSE = 8.92°) but failed at 30° separation.

**Conclusion:**

The phantom provides reliable repeatability for standard DTI metrics and demonstrates utility for quality assurance of advanced diffusion models with high angular resolution protocols.

## 1 Introduction

Diffusion MRI (dMRI) is a non‐invasive imaging technique that probes the microscopic movement of water molecules in biological tissues. Since water diffusion is influenced by structural barriers such as cell membranes, axonal fibers, and extracellular matrix components, dMRI can reveal important features and pathological changes that are not detectable with standard anatomical imaging [1]. It is widely used in neuroimaging to assess stroke [2, 3], traumatic brain injury [4], and neurodegenerative diseases [5, 6], among other conditions. Quantitative metrics such as apparent diffusion coefficient (ADC) and fractional anisotropy (FA) reflect tissue characteristics [7], enabling objective comparisons between patients and time points.

Despite its utility, dMRI presents significant challenges in standardization and reproducibility [8, 9, 10], particularly in regions with complex white matter architecture [11, 12, 13]. In the human brain, axonal fibers frequently cross, fan, branch, or merge, which results in differently oriented fiber bundles being present in the same voxel. Conventional models such as diffusion tensor imaging (DTI), which represent diffusion using a second‐order 3 × 3 tensor, assume a single dominant diffusion direction per voxel [7, 14]. This simplification leads to potential inaccuracies in regions with complex fiber geometry, often called the “crossing fiber problem” [15]. Studies estimate that 33% to 90% of white matter voxels exhibit such complexity and are affected by crossing‐fiber effects [16, 17, 18]. Compounding the issue, it has been established that dMRI signal intensity can differ even when imaging the same subject on the same scanner within a short time period, due to slow, progressive changes in the MRI baseline signal over time (i.e., scanner drift) [19, 20, 21]. dMRI's reliance on strong diffusion‐sensitizing gradients makes it especially susceptible to scanner‐induced instabilities, including gradient coil heating, which can alter gradient performance over time [19]. The use of echo‐planar imaging (EPI), while enabling rapid acquisition, further exacerbates sensitivity to B_0_ field inhomogeneities, susceptibility artifacts, and eddy current distortions, particularly in regions near air‐tissue interfaces.

To address the limitation of the DTI model, several advanced models have emerged. Diffusion Kurtosis Imaging (DKI) extends tensor models to capture non‐Gaussian diffusion, useful in regions with restricted or heterogeneous diffusion [22]. Constrained Spherical Deconvolution (CSD) estimates fiber orientation distribution functions (fODFs), allowing for multiple fiber populations per voxel [23, 24]. These techniques require more extensive data acquisition protocols with a greater number of diffusion directions and higher b‐values. While they may provide a more accurate characterization of complex fiber configurations, they are also more sensitive to noise and scanner instability, which raises questions about their reliability.

The goal of this work was to evaluate the repeatability of advanced higher‐order tensor diffusion metrics using a novel anisotropic diffusion phantom. The phantom is designed to mimic the scale and structure of brain tissue, particularly the hindered diffusion characteristics of white matter tracts, with known fiber‐crossing angles and fiber bundle dimensions. Recently, the phantom was evaluated for consistency in DTI‐derived metrics across multiple scanner platforms [25]. Here, the focus is on the repeatability of complex diffusion models, with specific emphasis on regions of crossing fibers. We adopt the same definitions for repeatability (consistency of measurement across time, under identical conditions) and reproducibility (consistency of measurement across time, under different conditions) as the Quantitative Imaging Biomarker Alliance (QIBA) framework [26, 27]. We use reliability as a broader term that encompasses both repeatability and reproducibility, which reflects the overall trustworthiness of a measurement across all conditions. The aim of this study was to establish baseline repeatability metrics for this phantom, which will help determine its suitability as a quality assurance tool for monitoring scanner performance over time, particularly for sites conducting longitudinal or multi‐site studies with advanced diffusion imaging protocols.

## 2 Methods

### 2.1 Phantom Design

**FIGURE 1:**
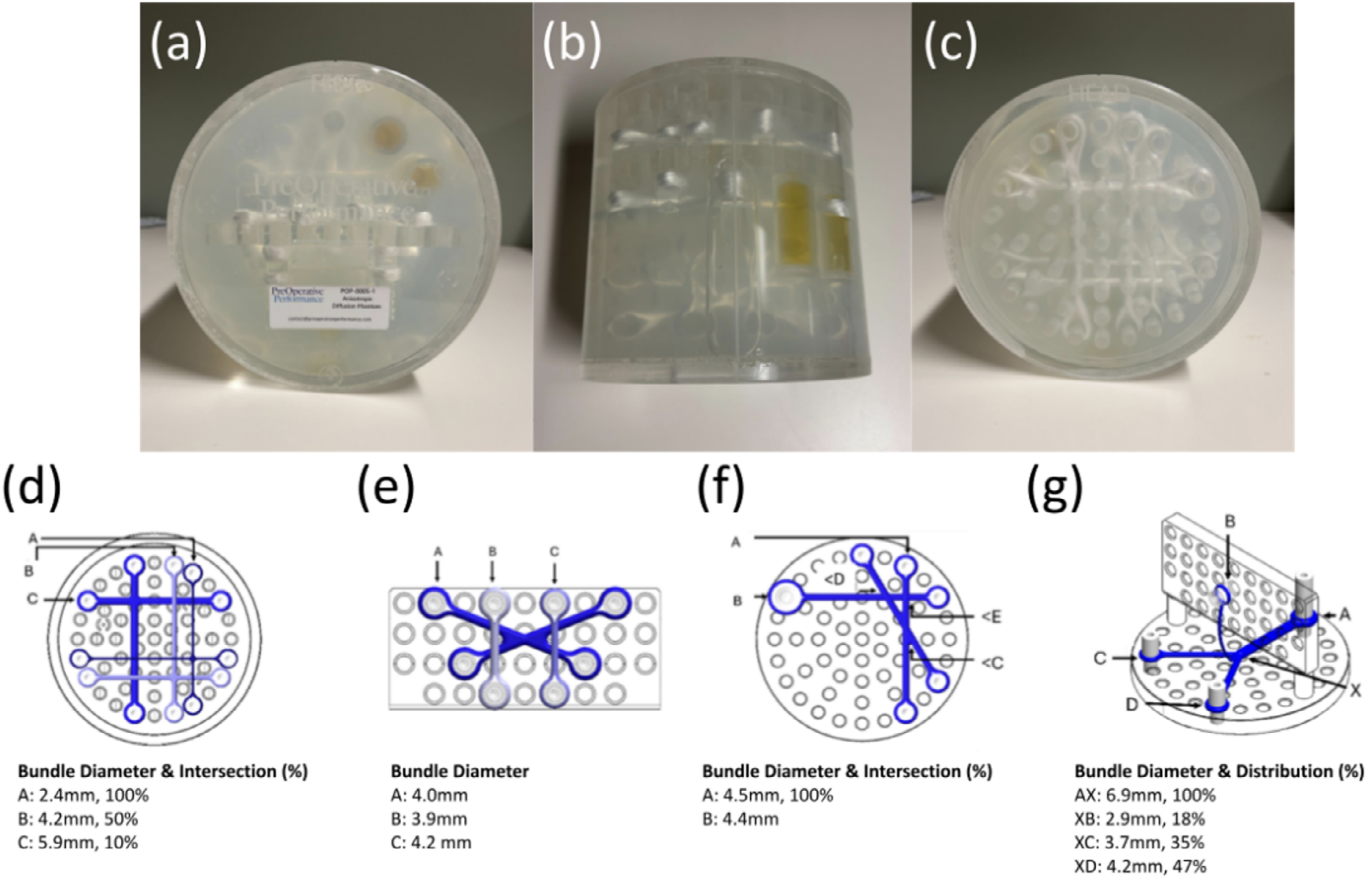
Photographs and schematics of the anisotropic diffusion phantom. (a–c) Photographs showing the front, lateral, and rear view. (d–f) Schematic diagrams of different module regions: (d) 90° crossings in the XY plane. (e) 45° crossing in the XZ plane. (f) Acute angle crossings (< C = 30°, < D = 60°, < E = 90°) in the XY plane. (g) Bifurcating module with out‐of‐plane element. Fiber AX splits into 3 smaller fiber bundles (two bundles in the XY plane, one bundle travels out‐of‐plane).

The phantom used throughout this study was designed and manufactured by PreOperative Performance (version POP‐0005‐001, Toronto, ON, Canada). The phantom consists of a sealed polymethyl methacrylate housing (height = 172 mm, outer diameter = 178 mm) filled with a polyvinyl alcohol (PVA) solution and doped with manganese chloride and a germicide preservative. Inside, arrays of fixed synthetic filaments are organized into different modules, including linear, bifurcating, and crossing fiber bundle geometries (Figure 1). The individual filaments have a diameter of 2 μm, and the fiber bundles have diameters ranging from 2.4 to 6.9 mm.

### 2.2 Data Acquisition

Data was collected using a 3.0 T GE Discovery MR 750 and a 32‐channel head coil (system software version 29.1, GE HealthCare, Milwaukee, WI). The phantom was imaged across 7 independent trials, acquired over two separate imaging sessions conducted 1 week apart, with complete removal and repositioning between each scan. By using a bubble level and aligning physical landmarks on the phantom housing with reference marks on the head coil, similar phantom positioning between trials was achieved. No rigid fixation device was used, as the goal was to assess repeatability under realistic quality assurance conditions where phantoms are routinely removed rather than permanently mounted. Prior to each trial, the phantom was removed from storage and allowed to equilibrate in the scanner room for ∼30 min before imaging.

Four different dMRI scans were performed: a 30‐direction DTI scan, a 60‐direction HARDI scan, a 90‐direction HARDI scan, and a 30‐direction multi‐shell DKI scan (acquisition parameters are detailed in Table 1). For each scan, 3 *b* = 0 s/mm^2^ volumes were acquired. ASSET parallel imaging (acceleration factor = 2) was enabled, and bandwidth was 2604 Hz/pixel for all diffusion scans. Multiband acceleration and fat saturation were not applied. Diffusion gradient tables were generated using the vendor‐provided scanner presets for each acquisition. In addition, a high‐resolution 3D T_1_‐weighted fast‐SPGR scan was obtained for each trial. All trials were conducted with consistent acquisition parameters and there were no changes to scanner hardware or software over the study.

**TABLE 1:** Summary table of dMRI scan acquisition parameters.

Protocol	Gradient directions	*b*‐values	TR/TE (ms)	Voxel size (mm)	FOV	Matrix size	Duration
DTI	30	0, 1000	7000/58.1	2.5 isotropic	24 cm	96 × 96	4:05
HARDI‐60	60	0, 1300	7000/61.6	2.5 isotropic	24 cm	96 × 96	7:35
HARDI‐90	90	0, 1300	7000/61.6	2.5 isotropic	24 cm	96 × 96	11:05
DKI	30	0, 250, 500, 750, 1000, 1500, 2000, 2500, 3000	8000/75.1	2.5 isotropic	24 cm	96 × 96	32:40

*Note*: The scans maintained constant spatial resolution (voxel size, FOV, matrix size) and slice prescription within trials. Total dMRI scan duration was 55 min, 25 s.

### 2.3 Data Analysis

For the bulk of data analysis, DIPY (version 1.9.0) was used. DIPY is an open‐source Python library for dMRI analysis, including reconstruction methods such as DTI, DKI, and CSD, as well as the ability to compute various diffusion‐related metrics [28]. In addition, the FMRIB Software Library (FSL) was used for specific preprocessing tasks and to model the DTI data. FSL is an open‐source software suite designed for preprocessing, analyzing, and visualizing MRI data [29].

Susceptibility‐induced distortion correction was performed using Polarity Reversed On Gradients to Reduce Susceptibility (PROGRES), available on the GE scanner. This application integrates reverse‐polarity phase encoding, motion correction, and eddy current correction within image reconstruction and has been shown to produce results comparable to FSL's *topup* [30]. Additional preprocessing steps included denoising using DIPY's Marchenko‐Pastur Principal Component Analysis (MP‐PCA) algorithm [31], followed by eddy current correction with FSL's *eddy* tool [32]. A binary mask was generated to isolate the phantom from the background signal and restrict modeling to the area of interest.

### 2.4 Data Modeling

DTI and HARDI data were processed using DIPY's *TensorModel*. To assess potential software‐related differences in tensor fitting, HARDI‐60 and HARDI‐90 acquisitions were additionally fitted using FSL's *dtifit*. Both approaches model the diffusion signal assuming Gaussian displacement of water molecules within each voxel.

(1) $$S\left(b\right)=S_{0}\exp\left(-\mathrm{bD}\right)$$

DKI data was processed using DIPY's *DiffusionKurtosisModel*, which extends the diffusion tensor model to capture non‐Gaussian effects [33]:

(2) $$\begin{array}{c} S\left(b\right)=S_{0}\exp\left(-\mathrm{bD}+\frac{1}{6}b^{2}D^{2}K\right) \end{array}$$

To resolve multiple fiber orientations for the DTI and HARDI data, CSD was applied using DIPY's *ConstrainedSphericalDeconvModel*. The response function, R(θ,ϕ), was estimated independently for each trial using the diffusion data from a region of the phantom known to contain a single, coherent fiber bundle and retaining voxels with FA > 0.7 [23]. The model fitting was performed using regularized spherical harmonics with order $l_{\max}=8$.

(3) S(θ,ϕ)=F(θ,ϕ)⊗R(θ)

After fitting each model, scalar diffusion metric maps were computed to perform quantitative comparisons across trials. For DTI/HARDI, this included fractional anisotropy (FA), mean diffusivity (MD), axial diffusivity (AD), and radial diffusivity (RD). For DKI, the derived metrics included kurtosis fractional anisotropy (KFA), mean kurtosis (MK), axial kurtosis (AK), and radial kurtosis (RK). For CSD, generalized fractional anisotropy (GFA) was calculated from the spherical harmonics.

### 2.5 Statistical Analysis

**FIGURE 2:**
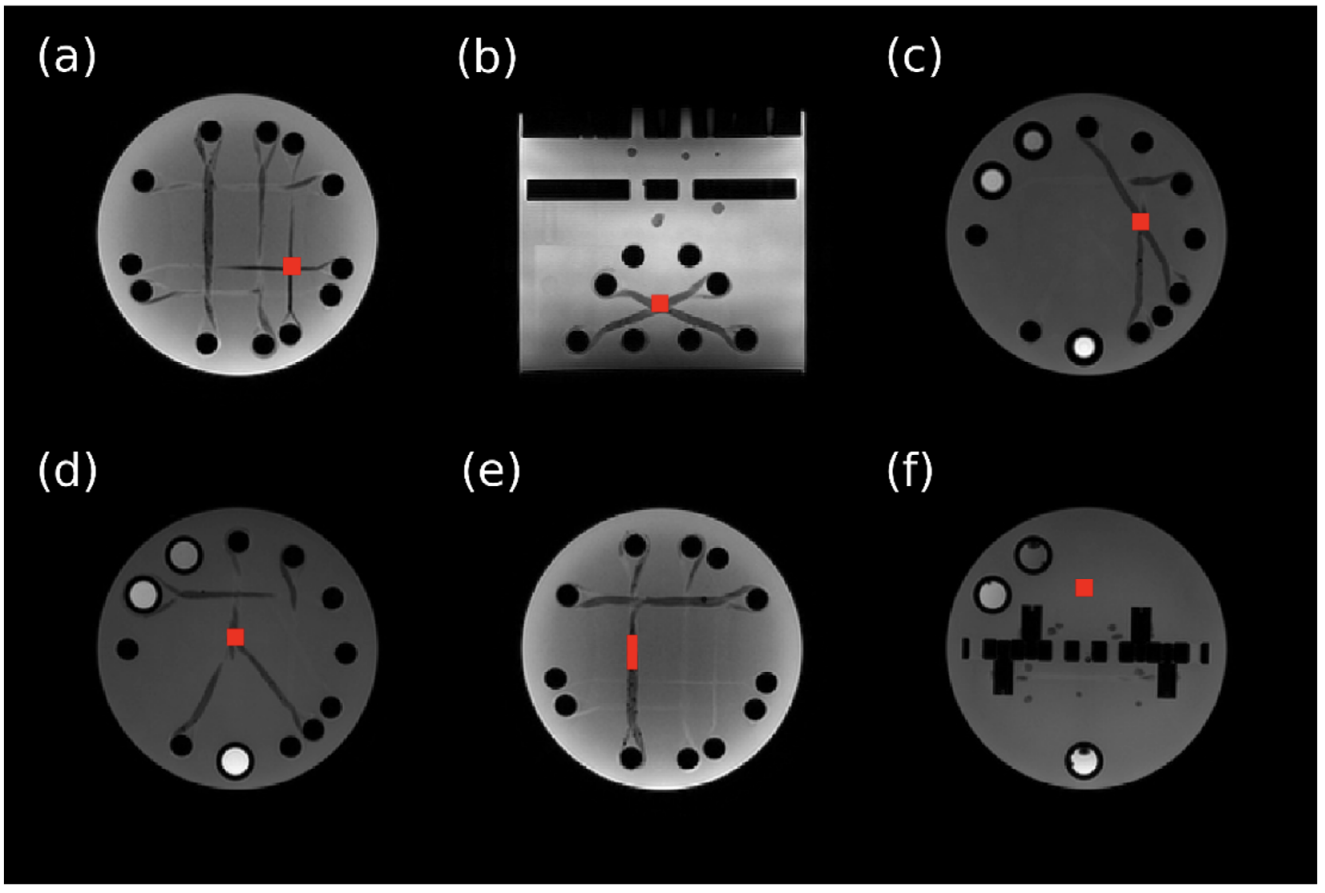
T_1_‐weighted images of the PreOperative Performance phantom showing the location of ROIs in red. (a) 90° intersection in the XY plane. Bundle diameters are 2.4 mm and the crossing is fully interwoven. (b) 45° intersection in the XZ plane, bundle diameter is 4.0 mm. (c) 30° intersection in the XY plane, bundle diameter is 4.5 mm. (d) Fiber splitting into three small fiber bundles. Two fibers split into the XY plane (pictured), and one fiber splits out‐of‐plane. Original fiber diameter is 6.9 mm and smaller fiber bundles are 2.9, 3.7, and 4.2 mm. (e) Simple linear fiber in the Y plane. Bundle diameter is 5.9 mm. (f) Isotropic area with no fibers present.

To assess repeatability of diffusion metrics across repeated phantom scans, statistical analyses were conducted on the scalar maps from each diffusion model. To enable voxel‐wise comparison across repeated scans, all scalar metric maps were co‐registered to a common space using FSL's *flirt* tool [34]. An affine 12‐parameter model was applied using the first trial as a reference volume. The same transformation was applied to all maps to maintain spatial consistency. For regions with complex fiber geometry, 3 × 3 × 3 voxel ROIs (yielding 27 values) were drawn (Figure 2a–d,f). For the simple linear fiber, a 2 × 7 × 2 voxel ROI (yielding 28 values) was used, oriented along the fiber length to accommodate the limited cross‐sectional dimensions of the linear segment while maximizing sampling along its principal direction (Figure 2e).

For each ROI and trial, the extracted voxel values from the ROI volume were averaged to provide 6 ROI values per trial. From these values, the across‐trial mean (μ) and across‐trial standard deviation (SD, σ) were computed. The coefficient of variation (CoV) was calculated to quantify the relative variability across repeated acquisitions:

(4) $$\begin{array}{c} \mathrm{CoV}=\frac{\sigma }{\mu }\times 100\% \end{array}$$

To summarize the overall repeatability for each diffusion metric and model, the average CoV across all ROIs was computed. The intraclass correlation coefficient (ICC) was also calculated using a two‐way mixed‐effect model, given that the 7 scan trials were performed on a fixed system under controlled conditions and not randomly selected as part of a larger set of trials. This is defined as:

(5) $$\begin{array}{c} \mathrm{ICC}\left(3,1\right)=\frac{MS_{\text{subjects}}-MS_{\text{error}}}{MS_{\text{subjects}}+\left(k-1\right)MS_{\text{error}}} \end{array}$$

where $MS_{\text{subjects}}$ is the mean square for the subjects (ROIs), $MS_{\text{error}}$ is the mean square error (residual variance), and k is the number of measurements (k=7). Differences between DIPY and FSL‐derived DTI metrics were evaluated using paired two‐tailed *t*‐tests (α=0.05).

**FIGURE 3:**
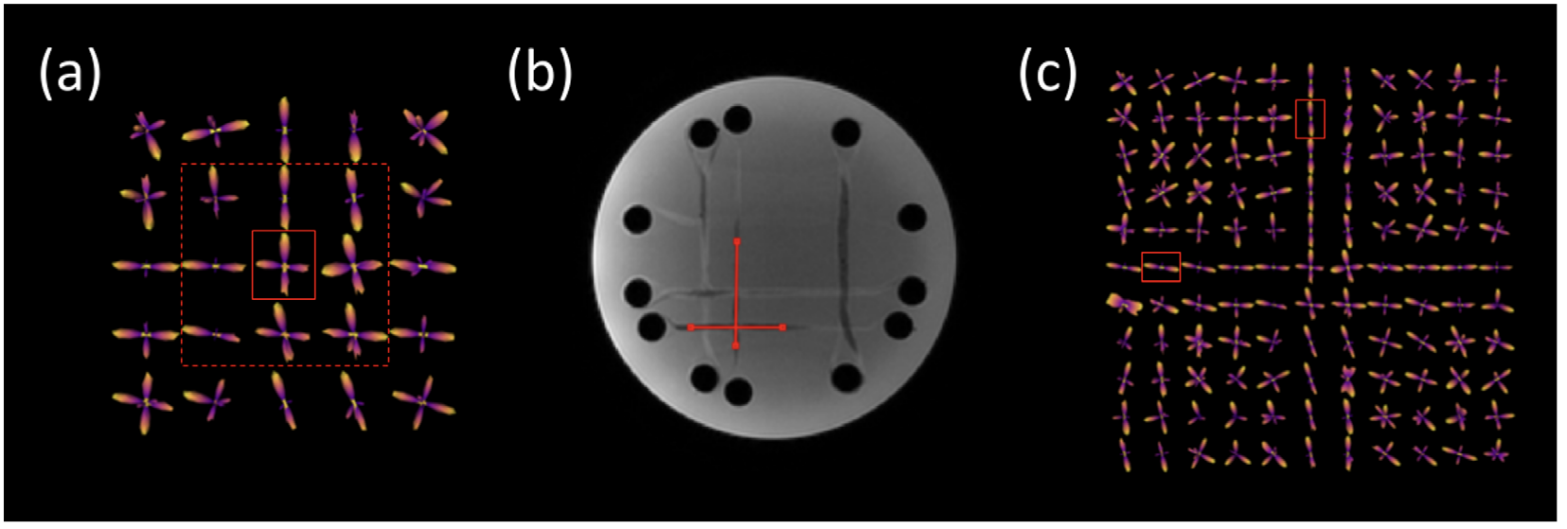
Qualitative assessment of crossing‐fiber repeatability in the phantom. (a) Angle between peaks calculated at crossing point voxel. Voxels were defined at the intersection (solid box) and extended by a ±1 voxel neighborhood to mitigate partial volume effects (dotted box). (b) Manual measurement of fiber crossings using high‐resolution T_1_‐weighted image. (c) fODFs along the approaching fiber bundles, where angular separations were calculated from single‐peak voxels to assess geometry relative to the intersection (red boxes).

To qualitatively assess CSD performance in resolving multiple fiber orientations, fODFs were generated from the 90‐direction HARDI data. Single voxels containing crossing fibers were identified in one trial, and corresponding locations in the remaining trials were obtained using *flirt* coregistration [34]. To account for partial volume effects introduced by repositioning between scans, a ±1 voxel neighborhood around each voxel identified through *flirt* was included (Figure 3a). This neighborhood was restricted to the eight adjacent voxels within the crossing plane (axial for ROI 1 and 3, coronal for ROI 2). Peak separation angles were calculated from fODF direction vectors and compared with the known fiber geometry. For each crossing configuration, the mean angle and root mean squared error (RMSE) were reported across trials.

To further characterize fiber crossing geometry, measurements were taken along bundles as they approached the intersection. Manual measurements were made on the T_1_‐weighted images to assess the accuracy of the fiber bundle placement compared with expected geometry (Figure 3b). Corresponding angular separations were also calculated from single‐peak fODF vectors on the approaching fiber bundle lengths (Figure 3c).

## 3 Results

### 3.1 Rank‐2 Diffusion Tensor Metrics

FA, MD, AD, and RD values were calculated for all 6 ROIs and across the three single‐shell acquisition schemes (DTI, HARDI‐60, and HARDI‐90). The average CoV across all 6 ROIs and ICC for the DIPY TensorModel fit are shown in Table 2. In general, these metrics showed high repeatability across all ROIs and scanning protocols. FA exhibited CoVs < 10% while MD, AD, and RD had CoVs < 3%. The overall trend shows the CoV decreased, and ICC increased when using HARDI acquisitions. All ICC values showed excellent reliability between scans (ICC > 0.9) while using the HARDI‐90 protocol. When compared with FSL's *dtifit* model, no statistical differences were found (Table S1).

**TABLE 2:** Summary of repeatability metrics (CoV and ICC) for DTI‐derived scalar measures across three single‐shell acquisition protocols (DTI, HARDI‐60, HARDI‐90) using DIPY's *TensorModel*.

	DTI		HARDI‐60		HARDI‐90	
	CoV %	ICC	CoV %	ICC	CoV %	ICC
FA	9.45 ± 4.16	0.9303	9.06 ± 4.04	0.9257	7.72 ± 3.68	0.9513
MD	2.06 ± 1.10	0.9336	1.95 ± 0.97	0.9401	1.89 ± 0.92	0.9474
AD	1.72 ± 0.95	0.8154	1.51 ± 0.71	0.8862	1.61 ± 0.71	0.9005
RD	2.40 ± 1.30	0.9431	2.32 ± 1.22	0.9543	2.45 ± 1.37	0.9474

### 3.2 Rank‐4 Diffusion Tensor Metrics

KFA, MK, AK, and RK values were calculated for all six ROIs using the multi‐shell DKI acquisition. Overall, the DKI‐derived metrics exhibited greater variability than their DTI counterparts. Among these, KFA demonstrated low CoV values (7.46%) and a high ICC (0.9161). In contrast, MK (CoV = 16.02%; ICC = 0.8312), AK (CoV = 12.89%; ICC = 0.9228), and RK (CoV = 19.24%; ICC = 0.8423) displayed higher variability across trials. Despite this increased variability, KFA and AK were classified as having excellent reliability based on their ICC values, whereas MK and RK demonstrated only good reliability.

### 3.3 Constrained Spherical Deconvolution

GFA showed a clear increase in reliability with angular resolution, as CoV and ICCs were 6.37% and 0.6920 (DTI), 4.07% and 0.8122 (HARDI‐60), and 4.16% and 0.8445 (HARDI‐90). Interestingly, CoV values remained low, even with only 30 gradient directions.

**FIGURE 4:**
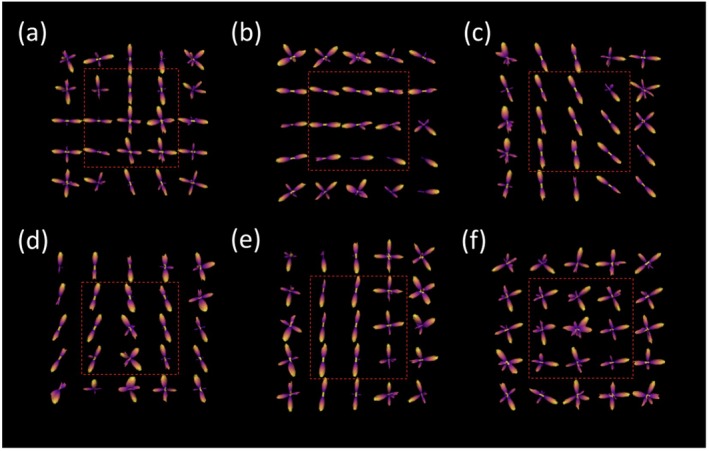
fODFs for the six ROIs given in Figure 1. Central slice is pictured (all axial except (b) which is coronal) and dotted box represents the 3 × 3 ROI boundary. (a) Two peaks at the 90° crossing. (b) Two peaks at the 45° crossing. (c) Single peak at the 30° crossing, as the model does not resolve both fibers. (d) Branching region with out‐of‐plane element shows three peaks, but orientations and angles are variable. (e) Single peak along the fiber. (f) Isotropic region with random orientations.

In crossing‐fiber regions, the ability of CSD to resolve multiple orientations varied with the separation angle (Figure 4a–c). At 30°, the model was not able to resolve the two fiber bundles and instead produced a single peak, indicating that the angular separation was below the effective resolution threshold. In contrast, the larger crossing angles consistently yielded two distinct peaks. For the 90° crossing, the mean separation was 87.10° with an RMSE of 3.49, closely matching the expected ground truth. For the 45° crossing, the mean separation was 50.07° with an RMSE of 8.92, indicating reduced accuracy and greater variability.

In the single fiber bundle region, a single dominant peak was consistently observed across all trials, with stable orientation (Figure 4e). The branching ROI showed less consistent results, with some trials producing multiple peaks and others only one or two (Figure 4d). In the isotropic region, fODFs did not exhibit a consistent orientation, and detected peaks appeared randomly distributed, consistent with the absence of directional structure (Figure 4f).

Manual angle measurements on the T_1_‐weighted image and CSD‐based measurements of fiber orientation as the bundles approached the crossing point were highly repeatable across trials and closely matched the expected geometry. For the 90° crossing, the average angular separation was 89.59° (RMSE = 0.47) for T_1_w and 89.39° (RMSE = 4.08) for CSD. For 45°, T_1_w averaged 47.26° (RMSE = 1.76) and CSD averaged 43.29° (RMSE = 3.61). For the 30° crossing, even though CSD failed to resolve the crossing fibers at the intersection point, the angle of the fiber bundles produced a repeatable measurement, with T_1_w measuring 29.72° (RMSE = 1.40) and CSD measuring 27.67° (RMSE = 3.63).

## 4 Discussion

This study demonstrated that the PreOperative Performance anisotropic diffusion phantom is a reliable and effective tool for evaluating the repeatability of advanced dMRI models under realistic QA conditions. Across seven independent trials, DTI‐derived scalar metrics showed excellent repeatability, with low CoV and high ICCs, particularly when HARDI acquisitions were used. Direct comparison of DTI tensor fitting using DIPY and FSL showed no statistically significant differences in ROI means or CoV, indicating that software implementation did not contribute meaningfully to metric variability. While DKI‐derived metrics exhibited greater variability (over 30% in some ROIs), KFA remained a stable and reliable measure across trials (< 10% in most regions). CSD‐based models accurately represent the phantom's crossing‐fiber configurations, with highly repeatable orientation estimates at 90° and 45°, demonstrating the phantom's value as a ground‐truth reference. Together, these findings establish the phantom as a practical and realistic QA tool for investigating advanced diffusion models and monitoring scanner performance over time.

The differences in repeatability across diffusion metrics and ROIs can be understood in context of their fiber orientation and underlying model assumptions. Tables S2–S7, found in Supporting Information, show mean and CoV values for all individual ROIs. For FA, it was noted that ROI 5 (linear) had the highest mean values, followed by ROI 3 (30° crossing), potentially due to the narrow 30° crossing angle leading to overlapping diffusion orientations and an artificially elevated principal axis. In contrast, ROI 1 (90°) had a much lower FA, indicating a more oblate tensor glyph and diminished anisotropy. This is also supported by ROI 1 having the lowest AD, since diffusion is suppressed along the principal axis due to the interweaving of orthogonal fibers. ROI 4 presented additional challenges, as it contains a small diameter fiber bundle (2.9 mm) and represents fibers that branch in a Y‐shaped configuration rather than crossing within a single plane. Together, these factors likely contributed to weak diffusion anisotropy and reduced signal‐to‐noise ratio, leading to elevated CoVs. ROI 6, the isotropic region, served as a low variance benchmark (CoVs < 1% for HARDI‐90 across all DTI‐derived metrics). For DKI metrics, no obvious trends emerged in the complex geometry fibers. ROI 5, despite being structurally simple, exhibited the most variable values and highest CoV. It showed the lowest MK (0.1333 × 10^−3^ mm^2^/s) but the highest CoV (30.73%), suggesting kurtosis metrics are less stable in simpler fiber geometries where non‐Gaussian effects are subtle. As expected, ROI 6, which contains no fibers, showed very low kurtosis values. This confirms that the Gaussian diffusion assumption holds well in isotropic regions, and the kurtosis model correctly reflects minimal microstructural complexity. CSD‐derived GFA showed clear improvement with increased angular resolution, demonstrating the model's dependence on sufficient directional sampling. Notably, ROI 3 (30° crossing) maintained relatively stable GFA values across protocols (CoV 3.06%–5.62%), but as demonstrated in the fODF analysis, this stability masks the model's inability to resolve the crossing at this acute angle.

In the crossing‐fiber regions, CSD failed to resolve discrete peaks at 30° separation but successfully detected crossings at larger angles. Accuracy declined at the 45° angle compared with 90° (RMSE 8.92 vs. 3.49). Tournier et al. demonstrated very similar degradation of angle accuracy using a fiber phantom [35] and Jeurissen et al. found the minimum angle that resolved reliably to be ∼55° in human brains [17]. This suggests that the challenges with 45° and 30° fiber separation represent a fundamental limitation of CSD rather than study‐specific factors. Future studies may benefit from higher b‐values and increased spherical harmonic orders to improve crossing fiber resolution, though this must be balanced against clinically relevant parameters and feasible scan times. A more repeatable measurement of fiber angle crossing was found to be measuring the angle of fiber bundles approaching the intersection point. Although this metric does not directly capture the angular separation within the crossing voxel, it provides a stable and repeatable estimate of the underlying fiber geometry and may serve as a complementary approach when evaluating tract orientation in regions of complex fiber architecture.

The repeatability results of this study align well with findings from earlier dMRI phantom research, particularly those using anisotropic phantoms designed to simulate white matter microstructure. Simard et al. conducted a reproducibility assessment using an earlier version of the PreOperative Performance phantom, focusing exclusively on DTI metrics (FA, MD, AD, RD) acquired across three different vendors and various motion probing gradient schemes [25]. Simard et al. found no statistically significant differences in diffusion metrics across vendor platforms or motion probing gradient tables. Other diffusion phantom reproducibility studies generally report low CoV for standard DTI metrics, with FA CoVs often below 5% in stable, linear fiber modules [36, 37, 38, 39, 40]. However, the extent of reproducibility depends heavily on phantom design. Phantoms with uniform fiber orientations and consistent packing tend to yield the lowest variability [36, 37]. In contrast, designs with heterogeneous features like variable fiber density or crossing angles often report higher CoV values, sometimes exceeding 10%–15% [41, 42]. This study found similar trends, with average FA CoV of ∼8% and even lower MD, AD, and RD CoVs (< 3%) with HARDI acquisitions.

Several studies have also extended phantom validation to higher‐order models such as DKI, showing that kurtosis metrics are more sensitive to noise and acquisition differences. Among DKI‐derived measures, MK, AK, and RK exhibit substantially higher CoVs than FA or MD, often ranging from 5% to 15%, with some values surpassing 20% in regions with crossing fibers or branching geometries [41, 43]. These trends reflect the additional instability introduced by estimating fourth‐order tensor components. Existing studies focus only on a subset of scalar measures or simpler fiber configurations, while this study examined four DKI‐derived metrics across multiple complex fiber geometries. To date, however, no studies have assessed the reproducibility of orientation‐resolved diffusion metrics, such as GFA, using physical phantoms.

Unlike the controlled environment of a phantom, in vivo variance is compounded by biological variability that depends on anatomical region, in addition to technical factors such as ROI size and the reproducibility of ROI placement [44, 45]. As such, aggregating variability metrics across human studies presents additional challenges. Zhong et al. reported global CoV for FA, MD, AD, and RD across 13 different ROIs as 9.1%, 5.3%, 3.6%, and 9.4% respectively [45]. Conversely, Palacios et al. reported lower variability, with CoVs across 26 ROIs of 4.1%, 2.44%, 3.41%, and 2.57%; however, they noted that smaller tracts tend to have higher variance, with CoVs reaching > 10% [10]. The CoVs presented here fall within these reported ranges, with FA similarly exhibiting the largest variability. Our results also indicated that ROI‐based variability for DKI metrics in the phantom reached up to 36% in specific ROIs. While this may appear high in isolation, it is consistent with, and often lower than, reported test–retest variability for DKI in the human brain. Kasa et al. reported global CoVs for MK ranging from ∼10% to 20%, AK from ∼5% to 10%, and RK from ∼15% to 35% depending on acquisition parameters [43]. In specific ROIs and datasets, they found CoV values exceeding 50%. These comparisons confirm that the phantom's variability reflects genuine technical challenges in acquisition and data modeling rather than phantom instability, validating its utility for isolating scanner performance and model fitting suitability from biological factors in quality assurance protocols.

**FIGURE 5:**
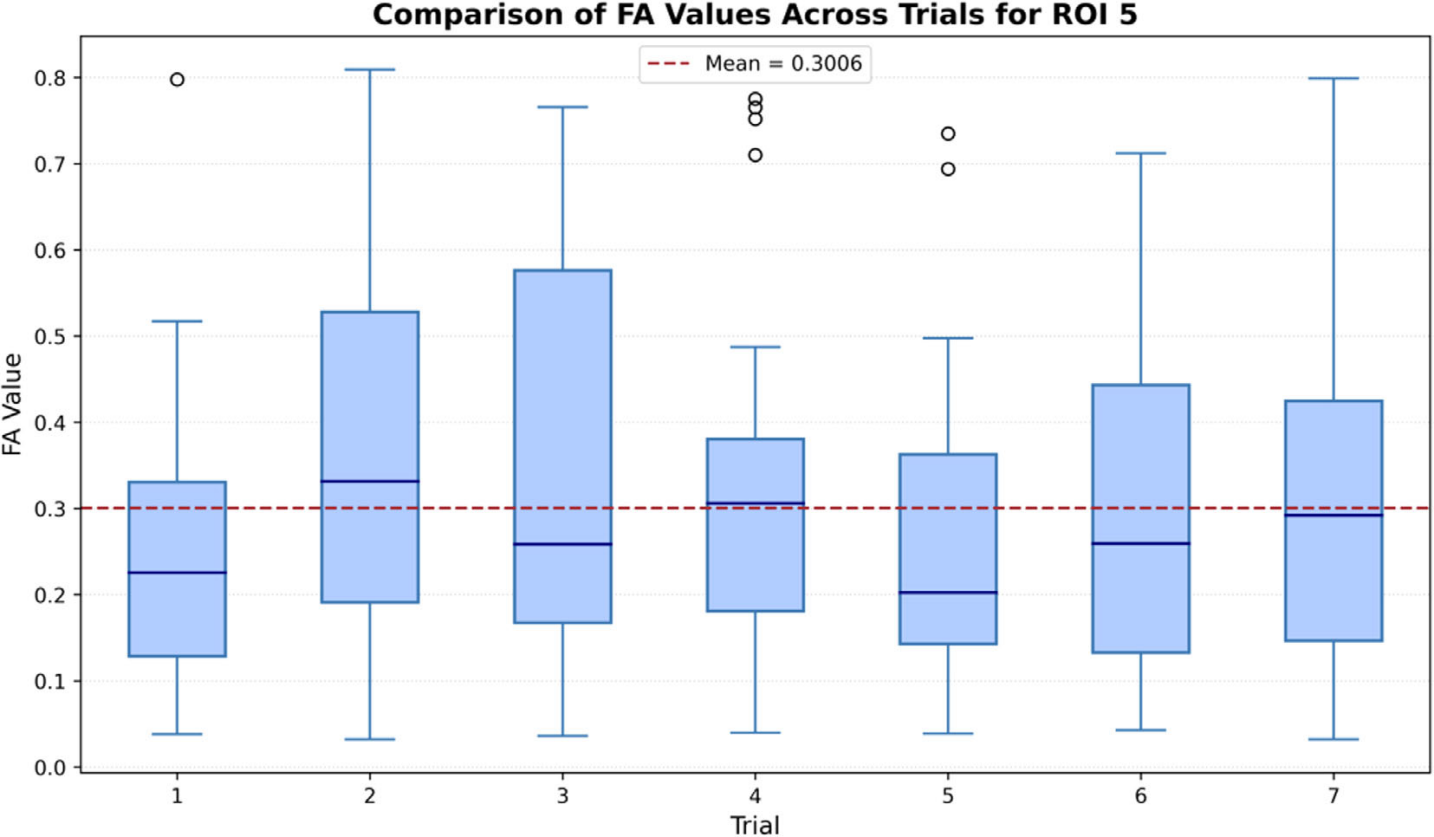
Boxplot shows the distribution of FA values measured in ROI 5 (simple linear fiber compartment) across all repeated imaging sessions. The red dashed line indicates the mean FA value (0.3006).

Several limitations should be acknowledged when interpreting these findings. All data was acquired on a single 3.0 T GE MRI system with consistent protocols, coil, and operator workflows. As such, the results represent intra‐scanner, intra‐protocol repeatability. This study also evaluated repeatability over a limited time span, and consequently, the long‐term stability over extended time scales (e.g., months to years) could not be assessed. While this design ensures internal consistency, it limits generalizability across protocols, vendors, field strengths, imaging centres, and longer‐term longitudinal time scales. Additionally, while the phantom was repositioned between trials, strict placement control (e.g., using a rigid holding structure) was not used. Although affine registration was applied during postprocessing, residual misalignment and partial volume effects could still affect voxel‐wise comparisons [46]. The 3 × 3 × 3 voxel ROI design reduced sensitivity to registration noise but inevitably included some isotropic background noise. Figure 5 shows the spread of FA values across all trials for ROI 5 (chosen because this is a simple linear fiber), with values ranging from 0.0322 to 0.809. The presence of low FA values within the data reflects partial‐volume effects and highlights the difficulty of isolating purely anisotropic signal in the small phantom compartments relative to the scan resolution (2.5 mm isotropic). In contrast, voxels positioned entirely within the fiber bundle exhibit high FA values (> 0.6), while those at the periphery contain mixtures of anisotropic fiber and isotropic background, producing intermediate values. Critically, this within‐ROI heterogeneity is consistently observed across trials.

Limitations also extend to the interpretation of repeatability metrics: CoV and ICC, while widely used to quantify repeatability, have their own assumptions and weaknesses. CoV is sensitive to the absolute scale of the metric, meaning that low‐magnitude values can appear highly variable even if the absolute change is small (as seen in RK and MK). ICC depends heavily on between‐subject variability, so models that blur regional distinctions (e.g., due to poor angular resolution) may show low ICCs even if they are stable within each ROI. Additionally, repeatability metrics do not reflect accuracy, as a highly repeatable model can consistently misrepresent the truth. Without ground truth measurements, the absolute error of each model remains unknown. This limits the ability to interpret whether variability is due to model limitations, measurement noise, or differences in the phantom metrics over time.

The current study was intentionally limited to a single scanner and fixed phantom geometry to isolate model‐specific effects. Future studies that extend the scope to assess inter‐scanner and inter‐vendor variability are a natural next step. MRI vendors attempt to harmonize acquisition protocols across platforms, but meaningful differences remain in gradient hardware, diffusion encoding schemes, RF coil designs, and image reconstruction algorithms [25]. Including scanners from multiple vendors (i.e., Siemens, Philips, Canon, etc.) would allow for a more comprehensive evaluation of reproducibility across imaging systems and could inform the development of vendor‐agnostic QA standards. In addition to vendor‐related factors, the choice of diffusion model itself plays an important role in the observed variability of derived metrics. The present work focused on DTI, DKI, and CSD to represent commonly used clinical and research‐oriented approaches, however, alternative modeling frameworks may offer improved stability in phantom‐based assessments. For instance, mean signal diffusion kurtosis imaging (MSDKI) has shown promise in reducing variability in kurtosis estimates by powder‐averaging signals across orientations [33, 47]. Furthermore, other high‐angular resolution techniques such as q‐ball imaging (QBI) or diffusion spectrum imaging (DSI) could be explored to see their performance in resolving complex fiber geometries [28, 35, 48]. Another important factor warranting systematic investigation is temperature dependence, since the rate of diffusion is dependent on temperature, affecting dMRI metrics [49]. In this study, the phantom was scanned under controlled room‐temperature conditions, but small deviations in thermal equilibrium may influence diffusivity and kurtosis estimates. For multisite studies, room temperature may fluctuate by several degrees [50], so characterizing the effect of ambient temperature could help guide best practices for using the phantom.

## 5 Conclusion

This study demonstrated that the PreOperative Performance anisotropic phantom is a reliable and effective tool for evaluating the repeatability of advanced dMRI metrics. Despite differences in model assumptions and sensitivities, the phantom supported consistent measurements across a range of fiber geometries, particularly under high angular resolution acquisitions. As advanced diffusion imaging continues to expand in clinical and research settings, standardized physical phantoms will be essential for quality assurance, protocol optimization, and cross‐site harmonization. Overall, this work establishes the phantom as a practical benchmark for diffusion metric stability and provides a foundation for future reproducibility studies across scanners, vendors, and acquisition strategies.

## Funding

This work was supported by Southern Ontario Pharmaceutical and Health Innovation Ecosystem (SOPHIE).

## Conflicts of Interest

Michael Noseworthy is co‐founder and CEO of TBIfinder Inc., a data analytics company focused on brain injury. Fergal Kerins is the founder and CEO of PreOperative Performance, and Sofia Chavez is a full time employee. PreOperative Performance provided a portion of the funding to support this project.

## Supporting information


**Figure S1:** DTI–derived parameter maps from a single slice of the phantom. Top panel shows the principal diffusion direction map, where FA modulates brightness and colors indicate direction as follows: red, left–right; green, anterior–posterior; blue, superior–inferior. The middle left panel shows the scalar FA map, and the middle right panel shows the MD map. The bottom panels show AD (left) and RD (right) maps. Diffusivity maps are in units of mm^2^/s.
**Table S1:** Summary of repeatability metrics (CoV and ICC) for DTI‐derived scalar measures across two single‐shell acquisition protocols (HARDI‐60, HARDI‐90) using FSL's *dtifit*.
**Table S2:** Comparison of FA values across ROIs and scanning protocols.
**Table S3:** Comparison of MD values across ROIs and scanning protocols. Mean and SD values are reported in units of × 10^−3^ mm^2^/s.
**Table S4:** Comparison of AD values across ROIs and scanning protocols. Mean and SD values are reported in units of × 10^−3^ mm^2^/s.
**Table S5:** Comparison of RD values across ROIs and scanning protocols. Mean and SD values are reported in units of × 10^−3^ mm^2^/s.
**Table S6:** Comparison of kurtosis metrics across ROIs. Mean and SD values for MK, AK, and RK are reported in units of × 10^−3^ mm^2^/s.
**Table S7:** Comparison of GFA values across ROIs and scanning protocols.

## Data Availability

Original DICOM data and analysis code will be made available upon request to the corresponding author.
