# Supplementary material for: Assessing Measurement Repeatability of a Novel Anisotropic Phantom for Advanced Diffusion MRI Models
Source: Magn Reson Med. 2026 Mar 6;96(1):339–48. doi: 10.1002/mrm.70330 (PMC13156440; doi:10.1002/mrm.70330)
Supplement: Supplementary file 1 — Figure S1: DTI–derived parameter maps from a single slice of the phantom. Top panel shows the principal diffusion direction map, where FA modulates brightness and colors indicate direction as follows: red, left–right; green, anterior–posterior; blue, superior–inferior. The middle left panel shows the scalar FA map, and the middle right panel shows the MD map. The bottom panels show AD (left) and RD (right) maps. Diffusivity maps are in units of mm2/s. Table S1: Summary of repeatability metrics (CoV and ICC) for DTI‐derived scalar measures across two single‐shell acquisition protocols (HARDI‐60, HARDI‐90) using FSL's dtifit. Table S2: Comparison of FA values across ROIs and scanning protocols. Table S3: Comparison of MD values across ROIs and scanning protocols. Mean and SD values are reported in units of × 10−3 mm2/s. Table S4: Comparison of AD values across ROIs and scanning protocols. Mean and SD values are reported in units of × 10−3 mm2/s. Table S5: Comparison of RD values across ROIs and scanning protocols. Mean and SD values are reported in units of × 10−3 mm2/s. Table S6: Comparison of kurtosis metrics across ROIs. Mean and SD values for MK, AK, and RK are reported in units of × 10−3 mm2/s. Table S7: Comparison of GFA values across ROIs and scanning protocols. [file MRM-96-339-s001.docx]

**Supporting Information**:


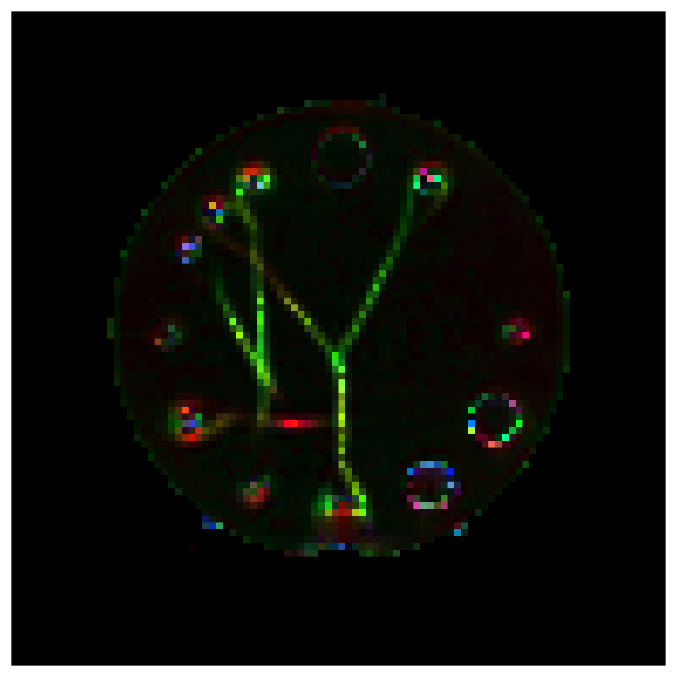


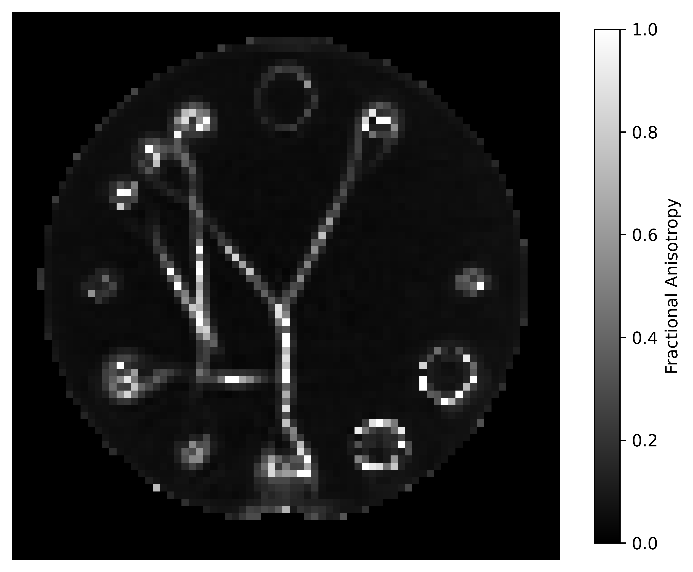

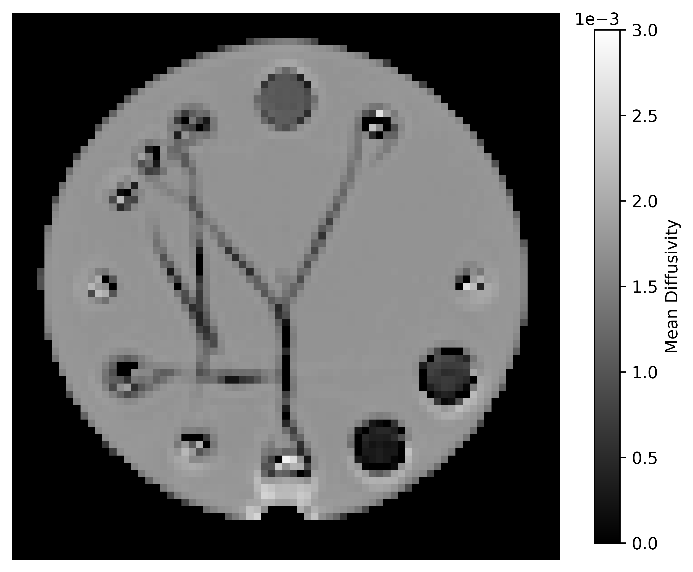


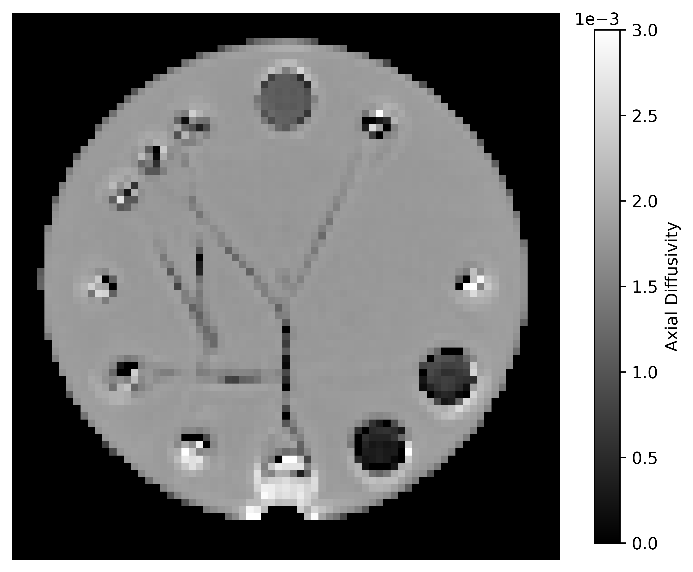

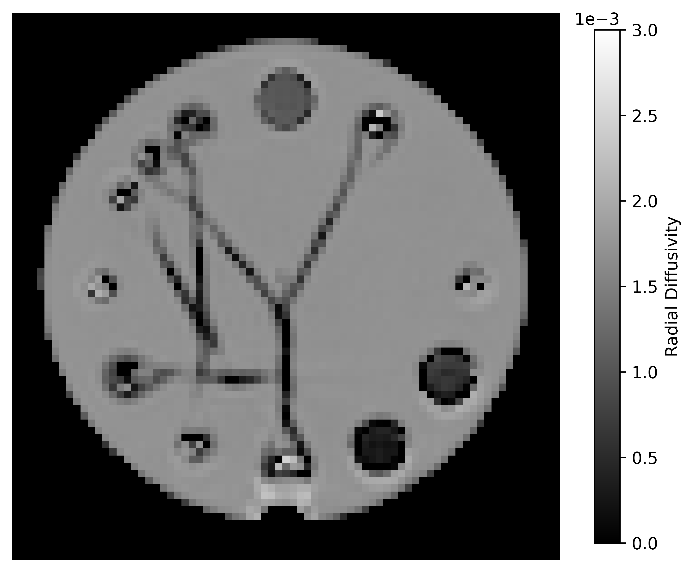


**Figure S1.** DTI–derived parameter maps from a single slice of the phantom. Top panel shows the principal diffusion direction map, where FA modulates brightness and colours indicate direction as follows: red, left-right; green, anterior-posterior; blue, superior-inferior. The middle left panel shows the scalar FA map, and the middle right panel shows the MD map. The bottom panels show AD (left) and RD (right) maps. Diffusivity maps are in units of mm^2^/s.

|  | **HARDI-60** | | **HARDI-90** | |
| --- | --- | --- | --- | --- |
|  | **CoV %** | **ICC** | **CoV %** | **ICC** |
| **FA** | 8.54 | 0.9478 | 8.16 | 0.9493 |
| **MD** | 2.45 | 0.9367 | 2.26 | 0.9378 |
| **AD** | 2.11 | 0.8506 | 1.92 | 0.8832 |
| **RD** | 2.93 | 0.9360 | 2.78 | 0.9409 |

**Table S1.** Summary of repeatability metrics (CoV and ICC) for DTI-derived scalar measures across two single-shell acquisition protocols (HARDI-60, HARDI-90) using FSL’s *dtifit*.

|  | **DTI** | | | **HARDI-60** | | | **HARDI-90** | | |
| --- | --- | --- | --- | --- | --- | --- | --- | --- | --- |
|  | **Mean** | **SD** | **CoV %** | **Mean** | **SD** | **CoV %** | **Mean** | **SD** | **CoV %** |
| **ROI 1** | 0.1685 | 0.0215 | 12.78 | 0.1662 | 0.0207 | 12.47 | 0.1684 | 0.0169 | 10.06 |
| **ROI 2** | 0.1654 | 0.0221 | 13.37 | 0.1625 | 0.0160 | 9.82 | 0.1593 | 0.0152 | 9.53 |
| **ROI 3** | 0.2422 | 0.0175 | 7.23 | 0.2493 | 0.0164 | 6.59 | 0.2507 | 0.0164 | 6.53 |
| **ROI 4** | 0.1735 | 0.0202 | 11.61 | 0.1778 | 0.0216 | 12.12 | 0.1781 | 0.0204 | 11.44 |
| **ROI 5** | 0.2921 | 0.0306 | 10.46 | 0.2871 | 0.0348 | 12.11 | 0.3006 | 0.0258 | 8.57 |
| **ROI 6** | 0.0543 | 0.0007 | 1.27 | 0.0490 | 0.0006 | 1.25 | 0.0455 | 0.0001 | 0.19 |

**Table S2.** Comparison of FA values across ROIs and scanning protocols.

|  | **DTI** | | | **HARDI-60** | | | **HARDI-90** | | |
| --- | --- | --- | --- | --- | --- | --- | --- | --- | --- |
|  | **Mean** | **SD** | **CoV %** | **Mean** | **SD** | **CoV %** | **Mean** | **SD** | **CoV %** |
| **ROI 1** | 1.4303 | 0.0304 | 2.12 | 1.4330 | 0.0299 | 2.09 | 1.4227 | 0.0303 | 2.13 |
| **ROI 2** | 1.4330 | 0.0531 | 3.70 | 1.4427 | 0.0437 | 3.03 | 1.4318 | 0.0420 | 2.93 |
| **ROI 3** | 1.3770 | 0.0259 | 1.88 | 1.3907 | 0.0254 | 1.82 | 1.3702 | 0.0236 | 1.72 |
| **ROI 4** | 1.4799 | 0.0461 | 3.11 | 1.4968 | 0.0479 | 3.20 | 1.4797 | 0.0445 | 3.01 |
| **ROI 5** | 1.5561 | 0.0159 | 1.02 | 1.5611 | 0.0154 | 0.99 | 1.5578 | 0.0162 | 1.04 |
| **ROI 6** | 1.7524 | 0.0092 | 0.52 | 1.7682 | 0.0102 | 0.57 | 1.7563 | 0.0085 | 0.48 |

**Table S3.** Comparison of MD values across ROIs and scanning protocols. Mean and SD values are reported in units of $\times$10^-3^ mm^2^/s.

|  | **DTI** | | | **HARDI-60** | | | **HARDI-90** | | |
| --- | --- | --- | --- | --- | --- | --- | --- | --- | --- |
|  | **Mean** | **SD** | **CoV %** | **Mean** | **SD** | **CoV %** | **Mean** | **SD** | **CoV %** |
| **ROI 1** | 1.6139 | 0.0420 | 2.60 | 1.6069 | 0.0415 | 2.58 | 1.6113 | 0.0455 | 2.82 |
| **ROI 2** | 1.6415 | 0.0529 | 3.22 | 1.6396 | 0.0365 | 2.23 | 1.6438 | 0.0320 | 1.95 |
| **ROI 3** | 1.6810 | 0.0176 | 1.04 | 1.6719 | 0.0203 | 1.21 | 1.6834 | 0.0187 | 1.11 |
| **ROI 4** | 1.7048 | 0.0314 | 1.84 | 1.7036 | 0.0249 | 1.46 | 1.7166 | 0.0319 | 1.86 |
| **ROI 5** | 1.7242 | 0.0191 | 1.11 | 1.7297 | 0.0192 | 1.11 | 1.7391 | 0.0246 | 1.41 |
| **ROI 6** | 1.8121 | 0.0090 | 0.50 | 1.8229 | 0.0018 | 0.46 | 1.8484 | 0.0103 | 0.56 |

**Table S4.** Comparison of AD values across ROIs and scanning protocols. Mean and SD values are reported in units of $\times$10^-3^ mm^2^/s.

|  | **DTI** | | | **HARDI-60** | | | **HARDI-90** | | |
| --- | --- | --- | --- | --- | --- | --- | --- | --- | --- |
|  | **Mean** | **SD** | **CoV %** | **Mean** | **SD** | **CoV %** | **Mean** | **SD** | **CoV %** |
| **ROI 1** | 1.3439 | 0.0284 | 2.12 | 1.3306 | 0.0306 | 2.30 | 1.3386 | 0.0297 | 2.22 |
| **ROI 2** | 1.3421 | 0.0518 | 3.86 | 1.3278 | 0.0469 | 3.53 | 1.3287 | 0.0564 | 4.25 |
| **ROI 3** | 1.2443 | 0.0313 | 2.51 | 1.2194 | 0.0277 | 2.27 | 1.2250 | 0.0306 | 2.50 |
| **ROI 4** | 1.3869 | 0.0578 | 4.17 | 1.3678 | 0.0554 | 4.05 | 1.3674 | 0.0553 | 4.05 |
| **ROI 5** | 1.4721 | 0.0170 | 1.15 | 1.4718 | 0.0182 | 1.24 | 1.4720 | 0.0168 | 1.14 |
| **ROI 6** | 1.7282 | 0.0102 | 0.59 | 1.7231 | 0.0087 | 0.50 | 1.7225 | 0.0093 | 0.54 |

**Table S5.** Comparison of RD values across ROIs and scanning protocols. Mean and SD values are reported in units of $\times$10^-3^ mm^2^/s.

|  | **KFA** | | | **MK** | | |
| --- | --- | --- | --- | --- | --- | --- |
|  | **Mean** | **SD** | **CoV %** | **Mean** | **SD** | **CoV %** |
| **ROI 1** | 0.4421 | 0.0490 | 11.09 | 0.2359 | 0.0301 | 12.74 |
| **ROI 2** | 0.4463 | 0.0416 | 9.31 | 0.2736 | 0.0490 | 17.90 |
| **ROI 3** | 0.4675 | 0.0379 | 8.10 | 0.2273 | 0.0375 | 16.50 |
| **ROI 4** | 0.4320 | 0.0184 | 4.26 | 0.2111 | 0.0186 | 8.81 |
| **ROI 5** | 0.5742 | 0.0570 | 9.92 | 0.1333 | 0.0410 | 30.73 |
| **ROI 6** | 0.1697 | 0.0036 | 2.10 | 0.0658 | 0.0062 | 9.43 |
|  |  |  |  |  |  |  |
|  | **AK** | | | **RK** | | |
|  | **Mean** | **SD** | **CoV %** | **Mean** | **SD** | **CoV %** |
| **ROI 1** | 0.2471 | 0.0304 | 12.28 | 0.2241 | 0.0367 | 16.38 |
| **ROI 2** | 0.3117 | 0.0284 | 9.10 | 0.2417 | 0.0470 | 19.47 |
| **ROI 3** | 0.2287 | 0.0242 | 10.58 | 0.2486 | 0.0424 | 17.04 |
| **ROI 4** | 0.1796 | 0.0282 | 15.70 | 0.2494 | 0.0263 | 10.54 |
| **ROI 5** | 0.1613 | 0.0250 | 15.52 | 0.1221 | 0.0447 | 36.60 |
| **ROI 6** | 0.0548 | 0.0077 | 14.08 | 0.0407 | 0.0063 | 15.46 |

**Table S6.** Comparison of kurtosis metrics across ROIs. Mean and SD values for MK, AK, and RK are reported in units of $\times$10^-3^ mm^2^/s.

|  | **DTI** | | | **HARDI-60** | | | **HARDI-90** | | |
| --- | --- | --- | --- | --- | --- | --- | --- | --- | --- |
|  | **Mean** | **SD** | **CoV %** | **Mean** | **SD** | **CoV %** | **Mean** | **SD** | **CoV %** |
| **ROI 1** | 0.1539 | 0.0108 | 6.99 | 0.2112 | 0.0112 | 5.33 | 0.2216 | 0.0045 | 2.05 |
| **ROI 2** | 0.1768 | 0.0167 | 9.44 | 0.1844 | 0.0069 | 3.76 | 0.1857 | 0.0113 | 6.08 |
| **ROI 3** | 0.2026 | 0.0062 | 3.06 | 0.1675 | 0.0094 | 5.62 | 0.1716 | 0.0075 | 4.36 |
| **ROI 4** | 0.1968 | 0.0142 | 7.20 | 0.2071 | 0.0052 | 2.52 | 0.2003 | 0.0092 | 4.60 |
| **ROI 5** | 0.1449 | 0.0084 | 5.79 | 0.1433 | 0.0057 | 3.95 | 0.1421 | 0.0044 | 3.08 |
| **ROI 6** | 0.0300 | 0.0017 | 5.76 | 0.0299 | 0.0010 | 3.25 | 0.0305 | 0.0015 | 4.81 |

**Table S7.** Comparison of GFA values across ROIs and scanning protocols.
